# miR-142-3p Modulates Cell Invasion and Migration via PKM2-Mediated Aerobic Glycolysis in Colorectal Cancer

**DOI:** 10.1155/2021/9927720

**Published:** 2021-07-13

**Authors:** JunYu Ren, Wenliang Li, Guoqing Pan, Fengchang Huang, Jun Yang, Hongbin Zhang, Ruize Zhou, Ning Xu

**Affiliations:** ^1^Department of Oncological Surgery, First Affiliated Hospital of Kunming Medical University, Kunming, Yunnan, China; ^2^Department of Pathology, First Affiliated Hospital of Kunming Medical University, Kunming, Yunnan, China

## Abstract

Decreased expression of miR-142-3p was observed in human cancers. However, the function and mechanism of miR-142-3p in human colorectal cancer remain obscure. The expressions of miR-142-3p in human colorectal cancer tissues and cell lines were measured by RT-qPCR. The effects of miR-142-3p on cell invasion and migration were detected by transwell assays. The efficiency of aerobic glycolysis was determined by glucose consumption and lactate production. Dual-luciferase reporter assays were performed to confirm the correlation between miR-142-3p and pyruvate kinase isozyme M2 (PKM2). The level of PKM2 was assessed by western blotting. Our results showed that the expression of miR-142-3p was decreased both in human colorectal cancer tissues and in cells. Overexpression of miR-142-3p in cell line attenuated colorectal cancer cell invasion and migration. About the underlying mechanism, we found that miR-142-3p modulated aerobic glycolysis via targeting pyruvate kinase M2 (PKM2). In addition, we demonstrated PKM2 and PKM2-mediated aerobic glycolysis contributes to miR-142-3p-mediated colorectal cancer cell invasion and migration. Hence, these data suggested that miR-142-3p was a potential therapeutic target for the treatment of human colorectal cancer.

## 1. Introduction

Colorectal cancer (CRC) is a common neoplasm with an increased rate of morbidity and mortality, and it has become a predominant malignancy worldwide [1]. Although the diagnosis has been improved, the mortality of colorectal cancer is still high.

MicroRNAs (miRNAs) are a group of small noncoding RNAs that play crucial roles in human cancers [2]. Aberrant expression of miRNA is reported to be potential targets for cancer therapy including breast cancer [3], prostate cancer [4], and colorectal cancer [5]. It was reported that miR-142-3p functions as a tumor suppressor and is involved in cancer development. For example, miR-142-3p inhibits the proliferation and invasion of pancreatic cancer cells [6]. A clinical study indicated that miR-142-3p is downregulated in plasma of CRC patients [7]. Moreover, studies suggested miR-142-3p is significant CRC progression. Zhu et al. suggested that miR-142-3p suppresses CRC cell growth [8]. Shen et al. indicated that miR-142-3p inhibits CRC cell proliferation via cell cycle regulation [9].

Aerobic glycolysis is a hallmark of cancers [10]. Studies indicated that aerobic glycolysis was involved in cancer cell aggressive behavior [11, 12]. miRNAs are closely related to aerobic glycolysis. In liver cancer, miR-199a-5p negatively regulates aerobic glycolysis [13]. miR-135 inhibits glycolysis of pancreatic cancer [14]. Accumulating evidence demonstrated that the modulation of aerobic glycolysis by miRNA was achieved by targeting glycolysis-related enzymes including phosphofructokinase-1 (PFK1) [14], pyruvate dehydrogenase kinase 1 (PDK1) [15], hexokinase 2 (HK2) [13], and pyruvate kinase M2 (PKM2) [16]. It has been well characterized that PKM2 plays a significant role in cancer development via aerobic glycolysis [17]. Moreover, previous bioinformatics analysis suggested miR-142-3p could directly target PKM2. However, the correlation of miR-142-3p and PKM2 in CRC warrants further investigation.

Here, we showed that miR-142-3p was negative related to colorectal cancer cell invasion and migration. We further demonstrated PKM2, a critical enzyme for glycolytic flux, was a target gene of miR-142-3p. PKM2 and PKM2-regulated aerobic glycolysis were required for miR-142-3p-mediated cancer progression. Thus, all of these results indicated that miR-142-3p modulates cell invasion and migration via mediating aerobic glycolysis in colorectal cancer.

## 2. Materials and Methods

### 2.1. Patients and Samples

CRC tissue samples and paired adjacent noncancerous tissue samples that were 2 cm away from the lesion were collected from 36 patients from the First Affiliated Hospital of Kunming Medical University from March 2016 to January 2018. The age range of patients was 20–60 years old with the average age of 40.36 ± 11.27 years old. None of the patients had previously received radiotherapy or chemotherapy before surgery. Written informed consent was received from all participants. The study was approved by the Ethics Committee of Yunnan Cancer Hospital ((2017) Ethics L No. 3).

### 2.2. Cell Culture and Transfection

Human colonic epithelial cells (NCM460) and human colorectal cancer cells (HCT116 and SW480) were obtained from the American Type Culture Collection (ATCC). Cells were cultured in DMEM supplemented with 10% heat-inactivated FBS and 100 unit/mL penicillin-streptomycin at 37°C in a 5% CO_2_ atmosphere. For cell transfection, miR-142-3p mimic and miR-142-3p NC mimic were constructed by Guangzhou RiboBio Biotechnology Co., Ltd. (Guangzhou, China). 50 nM miR-142-3p NC mimic and 50 nM miR-142-3p mimic were transfected by lipofectamine 2000 transfection reagent (Invitrogen) according to the manufacturer's protocol.

### 2.3. Western Blotting Assay

Proteins were separated by sodium dodecyl sulfonate polyacrylamide gel electrophoresis (SDS-PAGE) and transferred to the PVDF membrane by wet transfer. After being blocked with 10% PBS-diluted skim milk, the membrane was incubated with primary antibodies against PKM2 (abcam, ab85555, 1 : 2000) and GAPDH (abcam, ab9485, 1 : 5000) at room temperature for 2 hours. After washing 3 times, the membrane was incubated with HRP-conjugated secondary antibodies at room temperature for 1 hour. For the quantification of western blot data, the ImageJ software was used to measure the intensity of each band.

### 2.4. Quantitative Real-Time PCR (RT-qPCR)

The expression of miR-142-3p was detected by qPCR. Tumor tissues were cut into small pieces and grind into powder in liquid nitrogen. The total small RNA extracted from tissues and cancer cells was isolated using TRizol reagent (Invitrogen), and 0.5 *μ*g RNA was used as a template for cDNA synthesis using the miRNA 1st Strand cDNA Synthesis Kit (Vazyme, MR101-01). qPCR analysis was performed using 2x SYBR Green PCR Mastermix (Solarbio). qPCR was performed at 95°C for 30 s, 40 cycles of 95°C for 5 s, and 60°C for 60 s. The following primer sequences were used: miR-142-3p forward primer: 5′-TGCGGTGTAGTGTTTCCTACTT-3′, reverse primer: 5′-CCAGTGCAGGGTCCGAGGT-3′; U6 forward primer: 5′-CTTCGGCAGCACATATAC-3′, reverse primer: 5′-GAACGCTTCACGAATTTGC-3′. GAPDH and U6 were used as an internal control. The 2^-*ΔΔ*Ct^ method was used for relative quantification of gene expression.

### 2.5. Cell Migration and Invasion Assay

Transwell assays were performed using Millipore transwell chambers (8 *μ*m pore size; cat. no. MCEP06H48; Millipore). Transfected HCT116 and SW480 cells (2 × 10^4^ cells/well) were seeded in the upper chambers of a 12-well plate (Corning, Inc.) in a 500 *μ*L serum-free medium, and another 500 *μ*L complete medium was added to the bottom chamber. The chamber was incubated for 24 h in a humidified 5% CO_2_ incubator at 37°C. At the end of incubation, cotton-tipped swabs were used to remove the nonmigratory and noninvading cells. For invasion assays, the upper chamber was coated with Matrigel (Millipore) for 30 min at 37°C. Then, the remaining cells were fixed with iced formaldehyde for 30 min and stained using 0.1% crystal violet at 37°C for 1 h. The images were captured using an inverted microscope (Olympus, at 100x magnification) and quantified by ImageJ 1.8.0 software (National Institutes of Health).

### 2.6. Dual-Luciferase Reporter Gene Assay

The binding of miR-142-3p and PKM2 was predicted by TargetScan 7.1 (http://www.targetscan.org/vert_71/). The wild-type (WT) or the mutant (MUT) 3′-untranslated region (3′-UTR) of PKM2 containing binding sites of miR-142-3p was cloned into the pmirGLO luciferase reporter vectors (LMAI Bio, LM-1439) using restriction endonucleases. H293T cells were cotransfected with wild-type/mutant-type 3′-UTR and miR-NC/miR-142-3p mimic using Lipofectamine 2000 reagents (Invitrogen, 11668019) for 48 h. Cells were lysed, and luciferase activity was assessed.

### 2.7. Immunohistochemistry

Human colorectal cancer tissues and adjacent tissues were fixed with 4% paraformaldehyde and dehydrated with ethanol. The samples were embedded in molten paraffin. Paraffin-embedded tissue was cut into sections, and these sections were deparaffinized and heated. Then, tissue sections were incubated with antibodies and detected by the DAB kit (DA1010, Solarbio).

### 2.8. Enzyme-Linked Immunosorbent Assay

The glucose and lactate levels were measured using a Glucose Assay Kit (K686-100, BioVision) and a Lactic Acid Assay Kit (BC2235, Solarbio), according to the manufacturer's protocol.

### 2.9. Statistical Analyses

All experiments were carried out at least three times; the representative images were shown. All quantitative data were presented as the mean ± SEM. Student's *t*-test was used to analyze the differences between the two groups. One-way ANOVA with Tukey's test was used to analyze the differences between multiple groups. *P* value < 0.05 was defined as statistically significant.

## 3. Results

### 3.1. miR-142-3p Overexpression Attenuates Cell Invasion and Migration of CRC Cells

**Figure 1:**
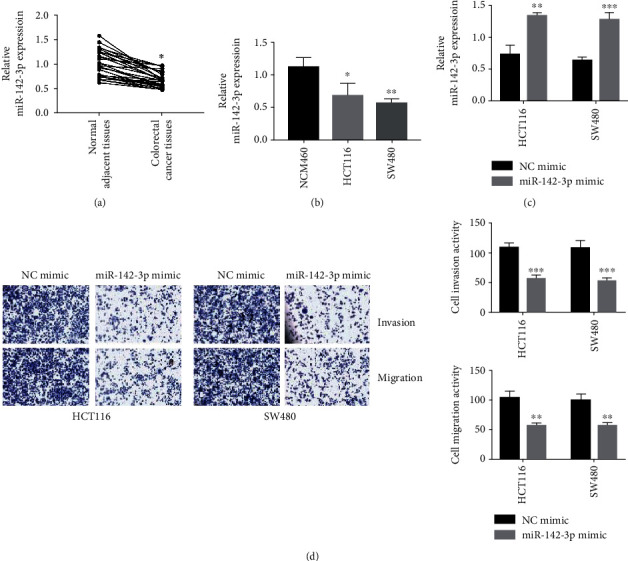
Aberrant expression of miR-142-3p in human colorectal cancer tissues and cell lines. (a) miR-142-3p expression in human colorectal cancer tissues and normal adjacent tissues was determined by RT-qPCR. Data were presented as mean ± SEM. *P* = 0.0142. (b) miR-142-3p expression was determined by RT-qPCR in colorectal cancer cells. Data were presented as mean ± SEM. *P* = 0.0344 and *P* = 0.0036. (c) HCT116 and T84 cells were transfected with miR-142-3p mimics and NC mimics. Relative miR-142-3p expressions were determined by RT-qPCR. Data were presented as mean ± SEM. *P* = 0.00217 and *P* = 0.00082. (d) Transwell assays were performed to determined invasion and migration of HCT116 and T84 cells. ^∗^*P* < 0.05, ^∗∗^*P* < 0.01, and ^∗∗∗^*P* < 0.001.

In order to determine the role of miR-142-3p in colorectal cancer, we first measured the expression level of miR-142-3p in human colorectal cancer tissues and corresponding normal adjacent tissues with RT-qPCR and observed that miR-142-3p was decreased in colorectal cancer tissues compared with normal adjacent tissues (Figure 1(a)). Moreover, the results of RT-qPCR indicated that the expression level of miR-142-3p in colorectal cancer cell lines was downregulated (Figure 1(b), ^∗^*P* < 0.05 and ^∗∗^*P* < 0.01). To further determine the function of miR-142-3p in colorectal cancer, we transfected HCT116 and SW480 cells with miR-142-3p mimics (Figure 1(c), ^∗∗^*P* < 0.01 and ^∗∗∗^*P* < 0.001). The results showed that overexpression of miR-142-3p impaired the invasion and migration in HCT116 and SW480 cells (Figure 1(d), ^∗∗^*P* < 0.01 and ^∗∗∗^*P* < 0.001). Thus, these data demonstrated that miR-142-3p was downregulated in colorectal cancer and might play a significant role in colorectal cancer development.

### 3.2. miR-142-3p Directly Targets the 3′-UTR of PKM2

**Figure 2:**
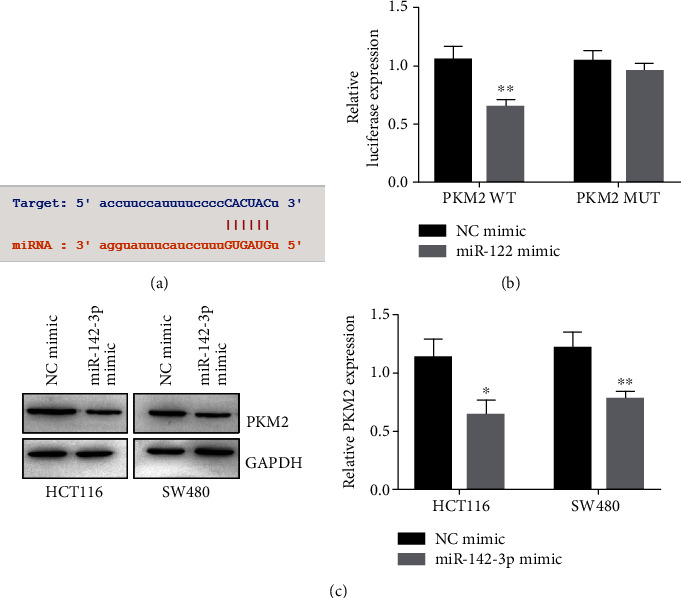
miR-142-3p directly targets the 3′-UTR of PKM2. (a) Binding sites of miR-142-3p and PKM2 were predicted by TargetScan. (b) Dual-luciferase reporter gene assay was performed to measure the relationship between miR-142-3p and PKM2. Data were presented as mean ± SEM. *P* = 0.0056. (c) PKM2 expressions were measured in HCT116 and T84 cells stably overexpressing miR-142-3p. Data were presented as mean ± SEM. *P* = 0.0135 and *P* = 0.007. ^∗^*P* < 0.05, ^∗∗^*P* < 0.01, and ^∗∗∗^*P* < 0.001.

miRNAs usually performing functions by regulating the expression of target genes. According to the TargetScan analysis, there is a binding site for miR-142-3p in the 3′-UTR of PKM2 (Figure 2(a)). To determine whether miR-142-3p could directly target PKM2, we performed dual-luciferase reporter assays. As shown in Figure 2(b), miR-142-3p decreased the luciferase activities of pGL4.49-PKM2-wt but had minimal effect on the luciferase activities of pGL4.49-PKM2-mut. In addition, western blot results indicated that overexpression of miR-142-3p inhibited the expression of PKM2 (Figure 2(c)). Collectively, these results showed that miR-142-3p negatively regulated PKM2 via binding to the 3′-UTR of PKM2 mRNA.

### 3.3. PKM2 Modulates Colorectal Cancer Cell Invasion and Migration via Aerobic Glycolysis Pathway

**Figure 3:**
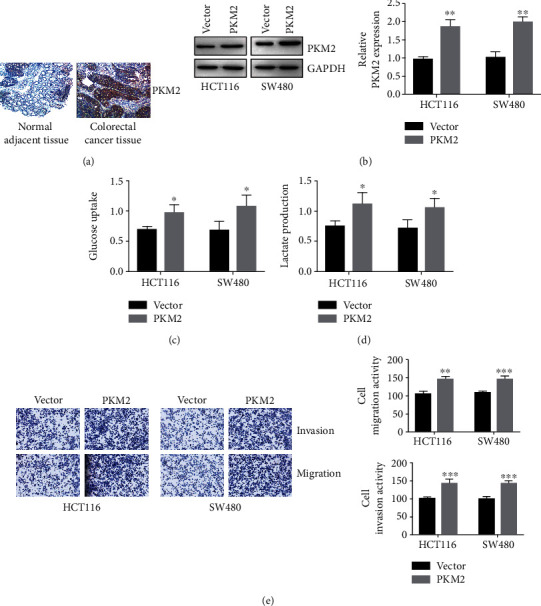
PKM2 modulates colorectal cancer cell invasion and migration via aerobic glycolysis pathway. (a) PKM2 expression in human colorectal cancer tissues and normal adjacent tissues was determined by immunohistochemistry. (b) PKM2 expressions were measured in HCT116 and T84 cells stably overexpressing PKM2. Data were presented as mean ± SEM. *P* = 0.002 and *P* = 0.0018. (c) Glucose uptake in HCT116 and T84 cells stably overexpressing PKM2 was measured by ELISA. Data were presented as mean ± SEM. *P* = 0.0258 and *P* = 0.0422. (d) Lactate production in HCT116 and T84 cells stably overexpressing PKM2 was measured by ELISA. Data were presented as mean ± SEM. *P* = 0.0368 and *P* = 0.0452. (e) Transwell assays were performed to determined invasion and migration of HCT116 and T84 cells stably overexpressing PKM2. ^∗^*P* < 0.05, ^∗∗^*P* < 0.01, and ^∗∗∗^*P* < 0.001.

As an important kinase in the aerobic glycolysis pathway, PKM2 was reported to modulate cancer development via regulating aerobic glycolysis. We first identified PKM2 expression in human CRC via immunohistochemistry and confirmed that PKM2 expression was upregulated in CRC (Figure 3(a)). To validate the role of PKM2 in colorectal cancer development, we overexpressed PKM2 in HCT116 and SW480 cells (Figure 3(b)). The results showed that overexpression of PKM2 resulted in increased cellular glucose uptake and lactate production, which suggested that overexpression of PKM2 in colorectal cancer cells enhanced aerobic glycolysis (Figures 3(c) and 3(d)). In order to identify the role of PKM2 and PKM2-mediated aerobic glycolysis in colorectal cancer development, transwell assays were performed. Overexpressed PKM2 promoted invasion and migration of HCT116 and SW480 cells (Figure 3(e)). Thus, these results suggested that PKM2 promoted invasion and migration of HCT116 and SW480 cells via aerobic glycolysis.

### 3.4. miR-142-3p Regulates Colorectal Cancer Cell Progression via PKM2-Mediated Glycolysis

**Figure 4:**
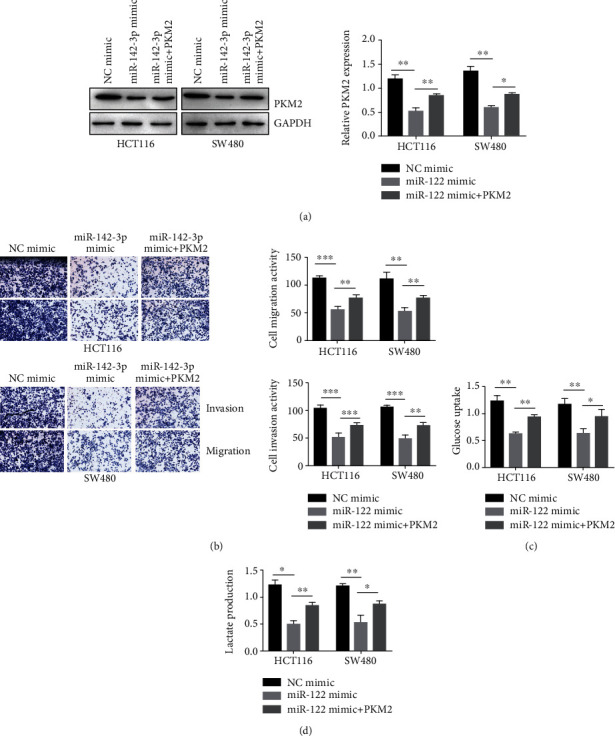
miR-142-3p regulates colorectal cancer cell progression via PKM2-mediated glycolysis. (a) PKM2 expression was measured in HCT116 and T84 cell stably overexpression miR-142-3p or/and PKM2. Data were presented as mean ± SEM. *P* = 0.0065, *P* = 0.002, *P* = 0.00745, and *P* = 0.0196. (b) Transwell assays were performed to determined invasion and migration in HCT116 and T84 cell stably overexpression miR-142-3p or/and PKM2. (c) Glucose uptake in HCT116 and T84 cell stably overexpression miR-142-3p or/and PKM2 was measured by ELISA. Data were presented as mean ± SEM. *P* = 0.0074, *P* = 0.00558, *P* = 0.00252, and *P* = 0.0299. (d) Lactate production in HCT116 and T84 cell stably overexpression miR-142-3p or/and PKM2 was measured by ELISA. Data were presented as mean ± SEM. *P* = 0.047, *P* = 0.0032, *P* = 0.00126, and *P* = 0.01839. ^∗^*P* < 0.05, ^∗∗^*P* < 0.01, and ^∗∗∗^*P* < 0.001.

Based on our previous results, we hypothesized that aberrant expression of miR-142-3p in colorectal cancer might modulate the PKM2-mediated aerobic glycolysis pathway and facilitated cancer development. To test this hypothesis, we transfected NC mimic or miR-142-3p mimic into HCT116 and SW480 cells with PKM2 overexpression (Figure 4(a), ^∗^*P* < 0.05 and ^∗∗^*P* < 0.01). Overexpression of PKM2 reversed the inhibition effect of miR-142-3p mimic on the invasion and migration in HCT116 and SW480 cells (Figure 4(b), ^∗∗^*P* < 0.01 and ^∗∗∗^*P* < 0.001). And then, we focused on the role of miR-142-3p in aerobic glycolysis in HCT116 and SW480 cells. As shown in Figures 4(c) and 4(d), miR-142-3p mimic transfection impaired glucose consumption and lactate production. In addition, overexpression of PKM2 reversed the inhibitory effect of miR-142-3p on glucose consumption and lactate production (Figures 4(c) and 4(d), ^∗^*P* < 0.05 and ^∗∗^*P* < 0.01). Thus, these data indicated that miR-142-3p regulated the invasion and migration of colorectal cancer cells via PKM2-mediated aerobic glycolysis.

## 4. Discussion

In the present study, we demonstrated that overexpression of miR-142-3p attenuated colorectal cancer cell invasion and migration. About the underlying mechanism, our results indicated that miR-142-3p could directly target 3′-UTR of PKM2 mRNA. PKM2 and PKM2-mediated aerobic glycolysis were required for miR-142-3p overexpression-induced inhibition of the invasion and migration in the colorectal cancer cell.

miRNAs are potential therapeutic targets for many cancers including colorectal cancer, and the mechanism of miRNAs involved in cancer progression is different. For example, cancer-associated fibroblast-derived exosomal miRNAs have a great influence on cancer progression [18]. miR-195-5p can modulate M2-like TAM polarization in colorectal cancer [19]. miRNA also involves in inflammation in colorectal cancer [20]. In addition, accumulating evidence indicated that miRNAs played a significant role in cancer cell metabolism. In colorectal cancer, miR-4999-5p reprograms glucose metabolism and affects cell proliferation [21]. miRNAs are reported to alter amino acid metabolism in colorectal cancer [22]. It was reported that miR-142-3p played a crucial role in colorectal cancer. The serum level of miR-142-3p is identified as a prognostic marker in colorectal cancer [23]. And the expression of miR-142-3p is related to CRC detection [24]. In the present study, we found that the level of miR-142-3p is downregulated in CRC tissues and cells. In order to confirm the function of miR-142-3p in colorectal cancer, we first overexpressed miR-142-3p in colorectal cancer cells. We observed overexpression of miR-142-3p attenuated the invasion and migration of colorectal cancer cells, which suggested that miR-142-3p could function as a tumor suppressor for colorectal cancer.

Aerobic glycolysis, a hallmark of cancer cells, has been well highlighted. The regulation of aerobic glycolysis by multiple factors has a strong effect on cancer progression [25]. The role of miR-142-3p in aerobic glycolysis has been well studied [26]. miRNAs usually modulate aerobic glycolysis via targeting related enzymes. For example, miR-142-3p suppresses aerobic glycolysis and hepatocellular cancer cell proliferation by targeting LDHA [26]. miR-214 promotes lung cancer cell proliferation and glycolysis by targeting HK2 and PKM2 [27]. It was reported that PKM2 is significant for aerobic glycolysis [28] and PKM2-mediated aerobic glycolysis is closely related to cancer progression [29]. Based on bioinformatics analysis, we found that PKM2 is one of the most significant targets of miR-142-3p. Our study further demonstrated that miR-142-3p could directly target PKM2 in colorectal cancer. Most importantly, PKM2 and PKM2-mediated aerobic glycolysis were required for miR-142-3p-regulated invasion and migration of colorectal cancer cell. Therefore, our findings indicated that miR-142-3p/PKM2 axis plays a vital role in regulating colorectal cancer metabolism and cancer cell malignancy.

## Data Availability

All data generated or analyzed during this study are included in this published article.
